# Comparison of All-Suture Anchors and Metal Anchors in Arthroscopic Rotator Cuff Repair: Short-Term Clinical Outcomes and Anchor Pullout Risk

**DOI:** 10.3390/jcm14082619

**Published:** 2025-04-11

**Authors:** Tolga Keçeci, Yusuf Polat, Abdullah Alper Şahin, Murat Alparslan, Serkan Sipahioğlu, Alper Çıraklı

**Affiliations:** Department of Orthopaedics, Ordu University Training and Research Hospital, 52200 Ordu, Türkiye; yusufpolat@odu.edu.tr (Y.P.); abdullahalpersahin@odu.edu.tr (A.A.Ş.); muratalparslan61@gmail.com (M.A.); serkansipahioglu@odu.edu.tr (S.S.); alperomu@gmail.com (A.Ç.)

**Keywords:** shoulder, suture anchor, rotator cuff, arthroscopy, single-row, double-row, complication, PROMS

## Abstract

**Objectives:** Metal anchors (MA), commonly used in the early stages of rotator cuff surgical treatment development, are associated with a high risk of complications, especially in osteoporotic bone. As an alternative to rigid anchors, all-suture anchors (ASA) have been introduced for the medial row, offering promising clinical outcomes and favorable biomechanical studies. We aimed to compare the clinical outcomes of MAs and ASAs in either single-row or in medial-row suture bridge techniques in arthroscopic rotator cuff repair. Our hypothesis was that in cases where ASA was used for at least 12 months of follow-up, more favorable results would be obtained as compared to rigid anchors, and intraoperative complications such as anchor pullout would be encountered less. **Methods:** In this retrospective cohort analysis, we reviewed patients who underwent arthroscopic rotator cuff repair between January 2020 and December 2022. Surgeries were performed by two senior surgeons in a single tertiary center. Patients who had undergone revision surgery, had a history of previous shoulder surgeries, had massive rotator cuff tears, and partial-thickness tears; or had concomitant subscapularis tears were excluded. Preoperative and postoperative scores, including Constant–Murley (CM), Disabilities of the Arm, Shoulder, and Hand (DASH), and visual analog scale (VAS), were compared. The minimum follow-up period was 12 months. Clinical assessment of shoulder range of motion included forward flexion, abduction, internal rotation, and external rotation. Intraoperative anchor-related complications were compared. All patients underwent the same surgical technique and postoperative rehabilitation protocol. **Results:** A total of 142 patients (89 females, 53 males; mean age: 57.4 years) were included in the study, with 67 patients in the ASA group and 75 in the MA group. The sex distribution and mean age were similar between groups. The ASA group had 15 traumatic tears, while the MA group had 13 (*p* < 0.05). The mean follow-up period was 21.6 months (range 12–40 months). Preoperative CM scores were statistically better in the ASA group, but this difference was not clinically relevant (*p* < 0.046). The mean CM score was 75.64, the mean DASH score was 8.57, and the mean VAS was 1.38 at the postoperative period in the MA group. The mean CM score was 78.40, the mean DASH score was 9.75, and VAS was 1.59 at the postoperative period in the ASA group. Seven cases experienced anchor pullout in the MA group, and thread breakage occurred in one patient of each group (*p* = 0.014). The mean age of the patients with anchor pullout was significantly higher (*p* = 0.002). This finding was not hypothesized in the initial study design but emerged during post-hoc analysis and highlights the importance of considering bone quality in elderly patients. **Conclusions:** The clinical outcomes of rotator cuff repairs using all-suture anchors or metal anchors are comparable. However, ASA use may offer an advantage in elderly patients by reducing the risk of anchor pullout. Further studies assessing tendon integrity and bone quality and incorporating long-term follow-up periods are recommended to support and validate the present findings.

## 1. Introduction

The management of symptomatic rotator cuff tears has evolved significantly, with treatment principles emphasizing a combination of conservative and surgical approaches tailored to individual patient needs. Non-operative management remains the first-line treatment for many patients, particularly those with partial tears or chronic conditions [1]. However, it is essential to recognize that, while conservative treatment can be effective, there is a risk of tear progression in some cases, particularly in larger or full-thickness tears [2].

As such, surgical options, including arthroscopic rotator cuff repair, are often considered when conservative measures fail [3]. Among the most common techniques are arthroscopic repairs, which can be performed using single-row or double-row configurations. The double-row technique, in particular, has gained popularity due to its biomechanical advantages, providing a larger contact area between the rotator cuff and the humeral footprint, which is believed to improve the stability of the repair and reduce the risk of re-tear [4].

The materials used in rotator cuff repair include various types of sutures and anchors, which can be made from absorbable or non-absorbable anchors. Bioabsorbable suture anchors have been introduced to minimize the risk of postoperative complications associated with permanent implants, such as osteolysis and cyst formation [5]. However, complications such as osteolysis, foreign body reaction, chondrolysis, and synovitis have been reported with bioabsorbable anchors [6]. To address these issues, all-suture anchors (ASAs) for the medial row have been introduced as an alternative. Positive clinical outcomes and favorable biomechanical study results have been reported [7,8,9,10,11,12,13,14,15].

In vitro studies have shown that ASAs have no toxic effect on human bone biology [16]. Biomechanical studies have presented that ASAs have mechanical strengths close to those of rigid anchors [17]. Radiologically examined case series demonstrated very little bone reaction or cyst formation in patients treated with ASAs [17].

The low-profile design of ASAs appears to be a significant advantage that will protect bone stock during insertion. ASAs exhibit similar resistance to failure and displacement loads as rigid anchors, e.g., in revision cases [18,19]. Unlike traditional anchors, the pullout strength of ASAs depends on the thickness of the humeral cortex [18,19]. It is advisable to evaluate cortical thickness in preoperative planning [19]. Other concerns include perianchor cyst formation and higher anchor pullout rates, but their impact on clinical outcomes is uncertain [8,13,14,15].

In response to the clinical challenges associated with rigid anchors, ASAs were introduced as an alternative anchor for the medial row. The literature has documented acceptable short-term follow-up outcomes with ASA in rotator cuff repair; however, there is a lack of comparative clinical studies involving different anchor types.

In this study, we aimed to compare the clinical results of metal anchors and ASAs in either single-row or in medial-row suture bridge techniques in arthroscopic rotator cuff repair. Our hypothesis was that, in cases where ASA was used for at least 12 months of follow-up, acceptable results would be obtained compared to rigid anchors, and intraoperative complications such as anchor pullout would be encountered less.

## 2. Materials and Methods

### 2.1. Patient Selection, Surgical Technique and Postoperative Rehabilitation

Ethical approval was obtained from the local institute’s ethics committee. Prospective records from a single tertiary center were retrospectively reviewed. Arthroscopic rotator cuff repairs performed by two surgeons between January 2020 and December 2022 were distinguished from the records. Inclusion criteria were determined as full-thickness tears, single-row repairs with metal anchors (MA) or ASA, suture bridge repairs using MA or ASA for the medial row, a minimum follow-up of 12 months, and being over 18 years of age. Patients with subscapularis tears, revision surgeries, massive tears (>5 cm), partial-thickness tears, and partial repairs were excluded from the study. In addition, patients presenting with neurological disorders affecting shoulder function, pseudoparalysis, and radiographic findings of proximal humeral migration and cuff tear arthropathy (Hamada grade ≥ 2) were also excluded. While comorbidities such as cervical spine pathologies, inflammatory arthritic diseases, diabetes mellitus, and thyroid disorders were evaluated during the preoperative period, they were not considered exclusion criteria. Given the advanced age of many patients within the cohort, these conditions were relatively prevalent. Excluding such cases would have resulted in a significant reduction in sample size and constrained the generalizability of the findings.

Preoperative magnetic resonance imaging (MRI) records of all patients were reviewed for tear size, tear retraction, and fatty infiltration of the muscles. Tear size was classified using T2-weighted sagittal oblique images as described by Cofield [20]: small, <1 cm; moderate, 1 to 3 cm; large, 3 to 5 cm; and massive, >5 cm. Tear retraction was assessed using the Patte classification [21] on T2-weighted coronal oblique images, which consists of 3 grades: (1) minimal retraction: the tear stump is lateral to the articular margin of the humeral head; (2) moderate retraction: the tear stump is between the lateral margin of the humeral head cartilage and the glenoid; and (3) severe retraction: the rotator cuff is retracted medially to the glenoid. Fatty infiltration of the rotator muscles was assessed using the Fuchs modification of the Goutallier classification [22,23]. In T1 sagittal oblique sections in supraspinatus, these classifications are the following: grade 1, no fatty infiltration in muscle or a small amount of fatty streak in muscle; grade 2, more muscle than fat; grade 3, equal amounts of muscle and fat or more fat than muscle.

All arthroscopic procedures were performed by two surgeons (TK and SS) under general anesthesia, with patients positioned in beach chair position. The choice of surgical approach depended on factors such as patient age, tear size, tendon retraction, and the extent of fatty degeneration in the muscle. Acromioplasty was performed on all patients. Double-row suture bridge repair was utilized in 33% of patients (47 cases), while single-row repair was employed in 67% of patients (95 cases) (Table 1).

For patients with biceps tendonitis, tenotomy was performed, whereas no additional procedure was conducted for those with healthy biceps. Tenodesis was not performed in any case.

For the single-row technique, 1 to 3 2.8 mm Q-Fix double-loaded ASAs (Smith & Nephew, Austin, TX, USA) were utilized depending on the width of the tear in ASA group. An accessory anchor portal was created to ensure an optimal insertion angle at a perpendicular angle to the bone. The suture limbs were passed through the tendon in a simple horizontal mattress configuration, and the knots were securely tied.

For the double-row suture bridge technique, suture limbs from the medial anchors were passed through the tendon at least 10 mm medial to the lateral edge using a FirstPass suture passer (Smith & Nephew, TX, USA) without knotting. These were fixed to the lateral row using 1 or 2 knotless 5 mm Artroline PEEK + titanium anchors (Artrotek, Adana, Türkiye) under appropriate tension. In some cases, a single lateral anchor was used, while in others, two anchors were applied in an “M” configuration depending on tear shape and size.

In the MA group, instead of ASA, 1 to 3 5 mm Artroline double loaded titanium anchors (Artrotek) were used according to the size of the tear using the same technique. In cases where intraoperative anchor pullout occurred, 6.5 mm cancellous metal anchors (Artrotek, Türkiye) were applied to achieve stronger fixation.

Postoperatively, all patients were instructed to wear a 30-degree padded abduction brace for 6 weeks. Gentle passive shoulder movements were initiated immediately after surgery, followed by active-assisted shoulder movements starting at 6 weeks. After the fourth month, patients were allowed to participate in simple activities. Patients were allowed to resume physical activities and sports after 9 months based on their recovery progress.

Preoperative range of motion (ROM), Constant–Murley (CM), Disabilities of the Arm, Shoulder, and Hand Questionnaire (DASH), and visual analog scale (VAS) scores were obtained from the medical records of patients who underwent surgery. Postoperative CM, DASH, and VAS scores at 12 months were retrieved from these records, and a preoperative-to-postoperative comparison was performed (mean follow-up: 21.6 months).

### 2.2. Statistical Analysis

Statistical analyses were performed using Jamovi Project version 2.3 (Sydney, Australia). Continuous data were described by mean and standard deviation, and categorical data were described by number and percentage. Differences in preoperative and postoperative values were assessed by paired *t* tests. Student *t* tests were used for parametric comparisons, and the Mann–Whitney U test was used for nonparametric comparison tests. Comparison of categorical parameters was performed using chi-square and Fisher exact tests. *p* < 0.05 was considered statistically significant for all comparisons.

## 3. Results

**Figure 1:**
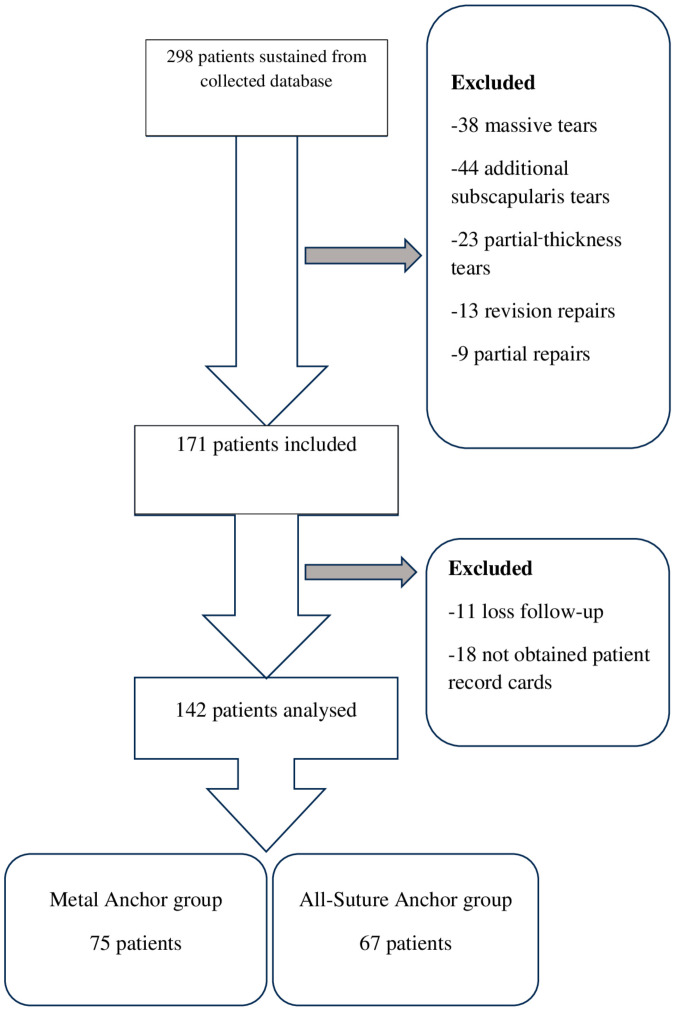
Flowchart of study groups.

The ASA group consisted of 67 patients, while the MA group included 75 patients who met the inclusion criteria (Figure 1). A total of 142 patients (89 females and 53 males) aged between 33 and 80 years with a mean age of 57.4 years were included in the study. Demographic characteristics, tear type, tear size, preoperative classification of supraspinatus fatty degeneration, repair technique, and the number of medial row anchors are summarized in Table 1. The gender distribution was similar between the two groups (female-to-male ratio: 51% to 49%). The number of traumatic rotator cuff tears was 15 in the ASA group and 13 in the MA group (*p* < 0.05).

The vast majority of patients included in this study presented with small to medium-sized rotator cuff tears. Most cases were classified as Grade I tear retraction according to Patte classification and exhibited Grade I fatty infiltration based on the Goutallier classification as shown in preoperative imaging. These selection criteria contributed to the homogeneity of the study population and allowed for a more accurate evaluation of short-term clinical outcomes following arthroscopic rotator cuff repair.

The rate of double-row repair was similar between the two groups (32–33%; *p* < 0.05). The mean follow-up period was 21.61 ± 7.56 months. Preoperative and postoperative CM, DASH scores, and ROM values for both groups are summarized in Table 2 and Table 3. In both groups, preoperative CM, DASH, and VAS values showed significant improvement at 12 months post-operation. The preoperative CM score was statistically higher in the ASA group compared to the MA group (ASA group: median = 47, IQR = 42–47; MA group: median = 45, IQR = 40–45; *p* = 0.046). However, this difference did not exceed the minimal clinically important difference (MCID) threshold of 10.4 points for CM score and thus was not considered clinically relevant [24]. No other statistically significant differences were found between the groups in terms of clinical scores or ROM measurements (*p* > 0.05).

In the ASA group, 39% of the patients were under the age of 55, while this proportion was 37% in the MA group. Among the total of 54 patients under 55 years of age, 25 individuals (46.3%) were actively engaged in physically demanding occupations. The remaining patients reported either occasional exercise or participation in low-intensity daily activities. Subgroup analyses showed no statistically significant differences in clinical outcomes—namely, CM, VAS, and DASH scores—when stratified by preoperative activity levels (*p* > 0.05). These findings suggest that activity level and younger age did not significantly influence short-term functional outcomes in this cohort.

Intraoperative anchor pullout complications occurred in 7 patients (9%) in the MA group but in none of the patients in the ASA group (*p* = 0.014, post-hoc power: 73%) (Table 4). Although not specified in the initial hypothesis, we observed during subgroup analysis that patients experiencing anchor pullout were significantly older than those without such complications (*p* = 0.002). This may suggest that older age could be a contributing factor to anchor-related failure. Demographics and clinical characteristics are presented in Table 5.

## 4. Discussion

This study shows that ASAs used in either single-row treatment or the medial row of double-row treatment for arthroscopic rotator cuff repair resulted in comparable clinical outcomes to metal anchors. Shoulder function improved in both groups independently of the anchor type following arthroscopic rotator cuff repair. At the final follow-up, there was no significant difference in functional outcomes between the two groups. However, we found more anchor pullout complications during surgery in the MA group. Also, the mean age of patients with anchor pullout was higher than the mean age of other patients.

Previous clinical studies have reported satisfactory outcomes with ASAs in arthroscopic rotator cuff repair [7,8,11,12,13,24]. Short-term results have been comparable to conventional anchors, with similar retear rates. In a cohort of 20 patients with 48 ASAs, VAS scores for pain improved from 6.88 to 2.12 at a minimum follow-up of 12 months. The mean Constant–Murley (CM) score in the same group was 79.05 [13]. Dhinsa et al. reported a mean CM score of 71.1 in patients who underwent double-row repair with ASAs at a minimum follow-up of 12 months, with intraoperative pullout occurring in two cases. Similarly, Memon et al. documented a mean CM score of 78.4 in 22 patients at a minimum follow-up of 12 months, with perianchor fluid collections observed in a small subset of patients, although these were not clinically relevant. Feldman et al. found similar ASES scores and VAS results between ASAs and metal anchors at a minimum follow-up of 2 years. The findings showed ASES scores of 89.6 and 88.8 in the solid and all-suture anchor groups, respectively. Loeb et al. reported reliable suture-bridge fixation using ASAs in 179 patients in a clinical study. In their study, the ASES scores at 24 months postoperatively were 87.3 ± 17.1. In this study, clinical outcomes were evaluated using the DASH score, which demonstrated significant improvement in both groups. By the end of 12 months, the mean CM and DASH scores indicated near-complete recovery in both groups. The findings had acceptable results supporting previous studies [9,11,12].

Prior biomechanical studies have demonstrated that ASAs provide sufficient stability and durability for rotator cuff repair [17,18,25,26,27,28], similar to their success in shoulder instability. Additionally, their implementation in smaller-diameter bone tunnels allows for the placement of a greater number of anchors within the footprint. This facilitates a neutralizing effect, contributing to a reduction in tensile stress on the sutures. Furthermore, the increased number of contact points with the cuff may potentially reduce the risk of retear [29]. Biomechanical studies have shown that in osteoporotic bone, all-suture anchors exhibit superior pullout strength [30] and increased stiffness relative to conventional solid anchors [31]. Based on these findings, it was suggested that ASAs may offer superior resistance to pullout stress in osteoporotic conditions due to their enhanced mechanical stability. Beyond the biomechanical advantages of ASA in rotator cuff repair, emerging evidence suggests that they may also promote tendon-to-bone healing. In a histological study conducted by Yu et al. using a rabbit model, a gradual integration of the tendon-bone interface was observed in the ASA group, indicating enhanced biological healing [32].

Although age and physical activity levels are often considered important predictors of postoperative functional recovery, our subgroup analysis did not demonstrate any significant differences in clinical outcomes (CM, VAS, and DASH scores) based on these factors. These findings align with certain previous studies suggesting that, in the context of standardized surgical techniques and rehabilitation protocols, the influence of baseline activity level and younger age on short-term outcomes may be limited [11]. The sample size of these subgroups may have been insufficient to detect subtle differences, and longer-term follow-up may be required to fully assess the impact of these variables on functional outcomes.

In our cohort, particularly among elderly patients, pullout was observed in seven cases within the MA group. This intraoperative complication, if encountered postoperatively, could lead to intra-articular loose body issues, such as chondrolysis. Anchor pullout during rotator cuff repair is influenced by a combination of factors, including bone quality, surgical technique, and the type of anchor used. Additionally, the technique employed during the procedure, such as the use of decortication, can significantly affect pullout strength. Decortication has been shown to decrease the pullout strength of suture anchors, indicating that careful consideration of surgical technique is essential to minimize the risk of failure [19,33]. Another key factor affecting anchor pullout is the quality of the bone at the site of anchor placement. Studies have shown that low bone mineral density (BMD) is associated with higher rates of anchor pullout, particularly in older patients or those with osteoporosis [34,35]. Notably, all intraoperative anchor pullout issues occurred in cases with 5.0 mm metal anchors in this study. In response, larger 6.5 mm cancellous anchors were applied intraoperatively to achieve secure fixation. This highlights the potential impact of anchor size on early fixation failure, especially in elderly patients with compromised bone quality. In environments where cortical bone is preserved, ASAs are less influenced by cancellous bone quality and are less likely to fail [19]. ASAs may be considered a reliable alternative to traditional anchors, as they can be safely used without the risk of pullout in cases with inadequate BMD.

In a study evaluating the biomechanical properties of ASAs, it was observed that no significant displacement occurred after cyclic loading with Q-Fix. While no pullout was reported, suture breakage was identified [26]. It is important to recognize that different ASA types may vary in their biomechanical performance. According to Ruder et al., Q-Fix anchors exhibited significantly less displacement (1.53 ± 1.00 mm after 200 cycles) and a higher ultimate failure load (191.3 ± 65.8 N) compared to other ASA models, which has been attributed to the ball-shaped configuration formed in the subcortical region during deployment [36]. In addition, comparative analyses with other anchor types, such as PEEK and biocomposite anchors, revealed no significant differences in displacement or ultimate failure load [18,25,27]. Similarly, in our study, no instances of pullout were observed in the medial row of the ASA group. On the other hand, pullout was observed in seven patients using the 5 mm metal anchor. In all these cases, a larger 6.5 mm cancellous metal anchor was required intraoperatively to ensure adequate fixation. These findings underscore the importance of bone quality and implant selection in achieving stable fixation during rotator cuff repair. Particularly in elderly patients or those suspected of having compromised bone quality, the use of all-suture anchors may offer a biomechanically safer and clinically effective alternative. Further studies with prospective designs and longer follow-up periods are warranted to validate these findings and guide optimal anchor selection in clinical practice.

The finding that older patients exhibited a higher rate of anchor pullout was not part of our original hypothesis or ethical committee application but emerged as a post-hoc observation during analysis. While this result should be interpreted with caution, it may indicate the importance of considering bone quality in anchor selection, especially in elderly populations. Although no distinguishing differences were observed in sex, tear size, degree of tendon retraction, or fatty infiltration grades between patients with and without anchor pullout, the limited number of cases precludes definitive statistical comparisons. However, based on these findings, it may be cautiously suggested that degenerative tear patterns and potentially poor bone quality associated with advanced age could be contributing factors in anchor pullout. The use of a single medial anchor, a smaller anchor diameter (5 mm), and the application of a single-row repair technique may have contributed to implant failure by increasing stress on the medial anchor. Future studies focusing on these specific factors may help to clarify the influence of bone quality and anchor type on anchor-related complications.

### Strengths and Limitations

This study has several limitations. Firstly, its retrospective nature introduces potential biases, including selection bias and reliance on previously recorded data. Secondly, while the surgical procedures were standardized, variations in tear characteristics and patient compliance with rehabilitation protocols may have influenced the outcomes. Additionally, the minimum follow-up of 12 months, while sufficient to assess short-term outcomes, may not capture long-term complications or retear rates. Excluding patients with subscapularis tears, revision surgeries, and massive retracted tears limits the generalizability of the findings to more complex rotator cuff pathologies.

Another important limitation is the absence of subgroup analysis based on repair technique (single-row vs. double-row/suture bridge). Although both techniques were used based on tear characteristics, biomechanical differences between constructs may have affected anchor performance. Stratified comparisons in future studies would help clarify the isolated effect of anchor type.

Additionally, no postoperative MRI evaluations were available. This limits the ability to assess tendon integrity, retear patterns, or anchor-related osteolysis. Similarly, BMD was not assessed, limiting our understanding of anchor pullout risk in osteoporotic patients.

No a priori power analysis was conducted due to the retrospective nature; post-hoc analysis revealed suboptimal power (0.07–0.78) for several comparisons. This indicates that some analyses may have been underpowered to detect small or moderate effect sizes, and thus, non-significant findings should be interpreted with caution.

Despite these limitations, the study has notable strengths. It provides a robust comparison of clinical outcomes between all-suture anchors and metal anchors in a well-defined cohort. The comprehensive assessment of preoperative imaging, range of motion, and patient-reported outcomes enhances the validity of the findings. Moreover, the standardized surgical and rehabilitation protocols ensure the consistency and reliability of the results. This study contributes valuable insights into the efficacy of ASA and MA in rotator cuff repair, informing clinical decision-making.

However, further prospective and long-term studies with larger cohorts are needed to confirm these results and refine surgical decision-making in rotator cuff repair, particularly in elderly populations.

## 5. Conclusions

This study demonstrated that arthroscopic rotator cuff repair using all-suture anchors resulted in clinical outcomes that were not statistically different from those of conventional metal anchors. No intraoperative anchor pullout was observed in the ASA group, while all pullout cases occurred in older patients in the MA group. Although the absence of statistical difference does not imply clinical equivalence, these findings suggest that ASAs may provide reliable fixation and could represent a safer alternative in elderly patients at higher risk of anchor failure due to compromised bone quality. Further prospective studies are needed to validate these results and guide optimal anchor selection in clinical practice.

## Figures and Tables

**Table 1:** Descriptive statistics for metal anchor and all-suture anchor groups.

	Groups (n/%)			
	MA	ASA	Total	*p* Value
Follow-up (months)	22.49 (28)	20.61 (22)	21.61 (24)	
Mean (Range)				0.144 ^a^
Age (years)				
Mean (range)	58.24 (41)	56.45 (47)	57.39 (47)	0.288 ^b^
Sex				
Female	45 (51)	44 (49)	89 (62)	0.485 ^c^
Male	30 (57)	23 (43)	53 (38)	
Dominance of Affected Side				
Dominant	66 (88)	53 (79)	119 (84)	
Non-dominant	9 (12)	14 (21)	23 (16)	0.151 ^c^
Tear Size-Cofield				
Small (<1 cm)	42 (56)	32 (48)	74 (52)	
Medium (1–3 cm)	25 (33)	29 (43)	54 (38)	0.475 ^c^
Large (3–5 cm)	8 (11)	6 (9)	14 (10)	
Tear Retraction-Patte				
Grade I	58 (77)	56 (84)	114 (80)	
Grade II	17 (23)	11 (16)	28 (20)	0.350 ^c^
Grade III	0 (0)	0 (0)	0(0)	
Tear Type				
Traumatic	13 (17)	15 (22)	28 (20)	0.450 ^c^
Degenerative	62 (83)	52 (78)	114 (80)	
Preoperative Fuchs Classification				
Grade I	61 (81)	58 (87)	119 (84)	
Grade II	11 (15)	7 (10)	18 (13)	0.706 ^d^
Grade III	3 (4)	2 (3)	5 (3)	
Amount of Medial Row Anchors				
1 anchor	51 (68)	46 (69)	97 (68)	
2 anchors	23 (31)	19 (28)	42 (30)	0.781 ^d^
3 anchors	1 (1)	2 (3)	3 (2)	
Repair Technique				
Single-row	51 (68)	45 (67)	96 (68)	
Double-row	24 (32)	22 (33)	46 (32)	0.915 ^c^
Total	75 (100)	67 (100)	142 (100)	

MA: Metal Anchor Group; ASA: All-suture Anchor Group; cm: Centimeter. ^a^ Welch *t* test; ^b^ Student *t* test; ^c^ Pearson chi-square test; ^d^ Fisher exact test.

**Table 2:** Means, standard deviations, and differences between mean test results of DASH, VAS, and CM in metal anchor and all-suture anchor group patients.

	Groups		
	MA, Mean (SD) [Median]	ASA, Mean (SD) [Median]	*p* Value ^a^
	75	67	
Preoperative Outcomes			
Preoperative CM	44.23 [45]	47.37 [47]	0.046 ^b^
Preoperative DASH	70.47 (10.59)	68.09 (9.65)	0.166
Preoperative VAS	7.51 [7.80]	7.22 [7.50]	0.155 ^b^
Postoperative outcomes			
Postoperative CM	75.64 (6.49)	78.40 (7.59)	0.065 ^b^
Postoperative DASH	8.57 (5.61)	9.75 (5.56)	0.214
Postoperative VAS	1.38 [1.50]	1.59 [1.70]	0.115 ^b^
Difference			
Difference DASH	61.89 (11.77)	58.34 (10.57)	0.062
Difference CM	31.41 (10.24)	31.03 (10.81)	0.829
Difference VAS	6.14 (1.69)	5.63 (1.60)	0.066

VAS: Visual Analog Scale; DASH: Disabilities of the Arm, Shoulder and Hand Questionnaire. CM: Constant–Murley score. MA: metal anchor. ASA: all-suture anchor. SD: standard deviation. ^a^ Student *t* test. ^b^ Mann–Whitney U test.

**Table 3:** Means, standard deviations, and differences between mean test results of range of motion in metal anchor and all-suture anchor group patients.

	Groups		
	MA, Mean (SD)	ASA, Mean (SD)	*p* Value ^a^
Preoperative ROM			
Elevation	130.5 (16.4)	134.6 (24.0)	0.243 ^b^
Abduction	110.7 (25.1)	113 (26.1)	0.567
Internal Rotation	3.73 (1.1)	3.96 (1.0)	0.215
External Rotation	52.5 (16.0)	55.4 (14.3)	0.269
Postoperative ROM			
Elevation	153.1 (19.0)	153.6 (20.9)	0.878
Abduction	141.9 (28.3)	148.1 (22.4)	0.149 ^b^
Internal Rotation	4.6 [4]	5.0 [5]	0.076 ^c^
External Rotation	66.5 (14.0)	70.5 (12.6)	0.082 ^b^
Difference ROM			
Elevation	22.5 (21.2)	19.0 (26.8)	0.384 ^b^
Abduction	31.2 (28.3)	34.9 (32.9)	0.473
Internal Rotation	0.9 (1.8)	1.0 (1.4)	0.541 ^b^
External Rotation	14.0 (12.6)	15.1 (16.1)	0.661

ROM: Range of Motion. MA: metal anchor. ASA: all-suture anchor. SD: standard deviation. ^a^ Student *t* test. ^b^ Welch *t* test. ^c^ Mann–Whitney U test.

**Table 4:** Anchor-related complications in groups.

		Groups		
	MA (%)	ASA (%)	Age/Mean	*p* Value
n	75	67		
No Complication	68 (91)	67(100)	56.80	
Intraoperative Anchor Pullout	7 (9)	0 (0)	68.86	0.014 ^a^
			0.002 ^b^	

MA: metal anchor; ASA: all-suture anchor. ^a^ Fisher’s exact test. ^b^ Student *t* test.

**Table 5:** Demographic and clinical characteristics of patients with anchor pullout in the MA group.

Patient	Sex	Age	Side	Dominant Side	Tear Type	Fuchs Grade	Patte Retraction Grade	Tear Size	Repair Type	Medial Row Anchor Number
1	Female	70	Right	Yes	Degenerative	Grade I	Grade I	Small	Single-row	1
2	Male	78	Right	Yes	Degenerative	Grade I	Grade I	Small	Single-row	1
3	Female	69	Right	Yes	Degenerative	Grade I	Grade I	Medium	Single-row	1
4	Male	70	Left	Yes	Degenerative	Grade I	Grade I	Medium	Single-row	1
5	Female	64	Right	Yes	Degenerative	Grade I	Grade I	Medium	Double-row	2
6	Female	70	Right	Yes	Degenerative	Grade III	Grade I	Medium	Single-row	2
7	Male	61	Right	Yes	Degenerative	Grade III	Grade II	Large	Single-row	2

## Data Availability

Data were obtained through the medical archive of Ordu University Training and Research Hospital and are unavailable due to privacy restrictions.
